# Using least median of squares for structural superposition of flexible proteins

**DOI:** 10.1186/1471-2105-10-29

**Published:** 2009-01-22

**Authors:** Yu-Shen Liu, Yi Fang, Karthik Ramani

**Affiliations:** 1 School of Mechanical Engineering, Purdue University, West Lafayette, IN 47907, USA; 2 School of Electrical Computer Engineering (by courtesy), Purdue University, West Lafayette, IN 47907, USA

## Abstract

**Background:**

The conventional superposition methods use an ordinary least squares (LS) fit for structural comparison of two different conformations of the same protein. The main problem of the LS fit that it is sensitive to outliers, i.e. large displacements of the original structures superimposed.

**Results:**

To overcome this problem, we present a new algorithm to overlap two protein conformations by their atomic coordinates using a robust statistics technique: least median of squares (LMS). In order to effectively approximate the LMS optimization, the forward search technique is utilized. Our algorithm can automatically detect and superimpose the rigid core regions of two conformations with small or large displacements. In contrast, most existing superposition techniques strongly depend on the initial LS estimating for the entire atom sets of proteins. They may fail on structural superposition of two conformations with large displacements. The presented LMS fit can be considered as an alternative and complementary tool for structural superposition.

**Conclusion:**

The proposed algorithm is robust and does not require any prior knowledge of the flexible regions. Furthermore, we show that the LMS fit can be extended to multiple level superposition between two conformations with several rigid domains. Our fit tool has produced successful superpositions when applied to proteins for which two conformations are known. The binary executable program for Windows platform, tested examples, and database are available from .

## Background

Protein flexibility is of great interest due to its essential role in various biological processes. The flexibility of dynamic regions allows a protein to assume multiple conformational states. Protein conformational changes play a critical role in biological functions such as ligand-protein and protein-protein interactions [1-5]. The rigid regions of the protein with highly structural stability will remain relatively unchanged between the multiple conformations in spite of any movement of the flexible regions [2-4]. In order to understand this kind of biological process, it is the first step to find out which regions keep the same and which change between two or multiple conformations. Structural superposition, defined as laying one molecule over the other by appropriate rotation and translation, is a common way to achieve that goal [2,6-8].

Superposition of molecular structures is an essential tool in structural bioinformatics and is used routinely in the fields of NMR, X-ray crystallography, protein folding, molecular dynamics, rational drug design and structural evolution [2,6-8]. The conventional superposition methods treat proteins as rigid bodies and use an ordinary *least squares *(LS) fit, in which the optimal rotations and translations are found by minimizing the root mean square deviation (RMSD) [9-13] between equivalent atom pairs. The LS fit for structural superposition of proteins is also called the RMSD fit. However, proteins are flexible molecules that undergo significant structural changes as a part of their normal function. When flexible molecules in different conformations are fitted to each other as rigid bodies, even strong structural similarity can be missed [14]. One main problem of the conventional LS fit is sensitive to local displacements [2,8,15,16]. In addition, most existing improvements of superposition, which strongly depend on the initial LS estimating for the entire atom sets of proteins [2,7,8,16-20], may fail on structural superposition of two conformations with large displacements. To correct these shortcomings of the conventional LS fit, we introduce a new fit algorithm based on the robust statistics techniques that will be explained later. Our method deals with the superposition of two conformations with small or large displacements without any prior knowledge of the flexible regions.

The heart of comparing two conformations of a protein is an appropriate overlay of the structures for visual inspection, where one protein is typically represented by its virtual *C*_*α *_atom chain of residues [2,13,21]. A relatively large number of protein structural comparison algorithms have been presented. They can be roughly categorized into two classes [22]: *structural superposition *and *structural alignment*.

In the structural superposition problems, protein structures are compared with a prior specified equivalence between pairs of residues (such an equivalence can be provided by sequence or threading algorithms, for example) [2,8,22]. The most commonly used superposition algorithm is the LS fit. The RMSD fit is a widely used algorithm to calculate the LS solution for evaluating the fit and quality of superposition [8]. The widely used algorithm to calculate the RMSD fit in matrix form was previously described by Kabsch [9-12]. This algorithm is the basis of most structural comparison methods that overlay molecules. Like most RMSD fitting procedures, the paper only superimposes the *C*_*α *_atoms, i.e. residues. Given two proteins composed of *N *atoms each, whose Cartesian coordinates are represented by an ordered set of points {**x**_1_, ..., **x**_*N*_} and a second set {**y**_1_, ..., **y**_*N*_}, respectively. The center of mass of both proteins are at the origin (it is trivial to translate any set of protein coordinates to accomplish this). The RMSD fit problem is then to find an orthogonal 3 × 3 matrix **U **by minimizing the following residual function:

(1) $$D_{rmsd}^{2}=\frac{1}{N}\sum_{i=1}^{N}{\left\|Ux_{i}-y_{i}\right\|}^{2}.$$

When $D_{rmsd}^{2}$ is a minimum, the square root of its value (i.e. *D*_*rmsd*_) becomes the minimal RMSD distance between two point sets. An alternative way to represent the two point sets uses two 3 × *N *matrices **X **and **Y**, where the *i*th column of **X **is the vector **x**_*i*_, and similarly for **Y**. The RMSD optimization consists of four steps [2,21,23]:

1. Compute a covariance matrix **R **= **XY**^*T*^.

2. Calculate the SVD (Singular Value Decomposition) of **R **= **VSW**^*T*^, where **V **and **W **are the matrices of left and right singular vectors, respectively, and **S **is the positive semidefinite diagonal matrix singular values of **R**.

3. Compute *χ *= sign(det(**R**)) = ± 1.

4. Calculate the rotation matrix **U **as

$$U=W\left(\begin{array}{ccc} 1 & 0 & 0\\ 0 & 1 & 0\\ 0 & 0 & \chi \end{array}\right)V^{T}.$$

An alternative RMSD fit approach uses a compact representation of rotational transformations called quaternions [9,10]. To make the RMSD effectively independent of the number of atoms, Maiorov et al. [13] have proposed a normalization mean. In addition, Wallin et al. [24] investigated and compared the properties of multiple distance measurements related to RMSD. More recently, Theobald et al. [8,16] applied the principle of maximum likelihood to the superposition problem by assuming a Gaussian distribution of the whole structures in the analysis. Additionally, algorithms based on multidimensional rotations and modified quaternions have been developed for structural superposition [25]. However, most existing improvements of superposition are based on the standard LS optimization. To overcome the disadvantages of the standard RMSD fit, some improvement algorithms, such as sieve-fit [19], fit-all [18], and HingeFind [20], are presented based on the iterative least-squares superposition by eliminations of atoms that lie far apart in the superposition. However, these algorithms depend on the initial RMSD fit for the entire atom sets of proteins, which may fail on structural superposition of two conformations with large displacements. Damm and Carlson [2] recently developed a Gaussian-weighted RMSD (wRMSD) fit, which makes use of a weight function for bounding the influence of atoms through an iterative LS fit. In order to overcome the effect of the initial RMSD fit, Damm and Carlson suggested large scaling factors for a global wRMSD fit code and they also recommended the local wRMSD fit on proteins with extreme structural changes. The wRMSD fit can achieve good results. In addition, several authors have reported some techniques for multiple structural superposition [8,16,26-29], where a simultaneous superposition could be employed to avoid biasing the superposition towards a specific (pivot) structure. We limit the study presented in this paper to pairwise structural superposition in term of fitting atomic coordinates of two conformations of the same protein.

Unlike structural superposition, structural alignment aims to compare a pair of structures, where the alignment between equivalent residues is not given prior. Therefore, an optimal sequence alignment needs to be identified, which has been shown to be NP-complete [30]. Many structural alignment methods, such as DALI [31] and CE [32], have been proposed to identify the defined best alignment. The general outputs of structural alignment are a superposition of equivalent atomic pairs and a minimal RMSD distance fitted between two structures. Recently, some methods consider the hinge regions for aligning the rigid subparts of the molecules [14,33]. Structural alignment is often composed of three steps: finding atom pair correspondence (alignment), superposition, and the RMSD calculation. Many structural alignment programs achieve both the correspondence and the superposition, simultaneously. Several papers [8,22] have clearly distinguished the difference between structural superposition and alignment. Although several recent works [25,34] are also named "*superposition*", they are actually related to structural alignment. These publications deal with different topics from our work. A review of many available methods for structural alignment is beyond the scope of this paper. The reader may consult Refs. [14,22,27,32,33] for detailed expositions.

The RMSD fit can be regarded as a LS fit [2,8], that finds a best rotation to fit a given atomic arrangement to approximately measured coordinates. The fit belonging to a statistical method is considered to be *robust *if it has a large *breakdown *point. A breakdown point might be loosely defined as the smallest percentage of outliers that can cause the estimator to take arbitrarily large aberrant values [35,36]. For instance, the breakdown point of the median of a set of values is 50% [36], whereas LS has a breakdown point of 0%. In this paper, we treat the displacements of two conformations of the same protein as *outlier*, i.e. location errors, during the fit process.

Several robust statistics methods have been applied to structural superposition of proteins [2,8]. For instance, Lesk presented the sieve-fit procedure [19] by eliminations of atoms that lie far apart in the initial fit. The algorithm is achieved through an iterative LS procedure as follows [37]. If the calculated RMSD between two point sets is larger than a threshold, the distances between the corresponding atoms in the sets are calculated. The atoms furthest apart are then removed from the original sets and the remaining atoms are superimposed again. This procedure is iterated with one pair of atoms being eliminated in each iteration, until the calculated RMSD is less than the threshold. The Lesk's sieve-fit procedure [19] is unsuitable for superposition between two conformations with multiple rigid domains. HingeFind, presented by Wriggers et al. [20,38], modified the sieve-fit routine so that the new atoms that are within tolerance distance are included in addition to the elimination of far apart atoms. Gerstein et al. [18] proposed the fit-all algorithm to classify the mechanism of domain rotation as hinge-like or shear-like. MolMovDB [17] used a modification of sieve-fit by stopping the procedure according to the domain size. These above algorithms can be regarded as the *backward methods *in statistical methods. The strategy of backward methods for fitting two point sets first fits to the entire points and then tries to remove bad points or weaken their effectiveness [35]. Unfortunately, as well-known in the statistics literature [35,39], some errors and outliers can influence the fitted model in the backward methods. The backward algorithms depend on the initial fit for the entire atom sets of proteins, which may fail upon structural superposition of two conformations with large displacements.

Damm and Carlson [2] used the wRMSD fit for superimposing two protein conformations in order to overcome the disadvantages of the LS fit. They also recognized that their method may yield poor results when the procedure starts with all the residue pairs for two significantly different structures (such as shifting the relative positions of two domains). Therefore, they presented the local wRMSD fit using an alternative starting procedure in a way similar to the forward regression spirit. The main difference between our work and wRMSD is the fitting optimization equations.

Recently, Fleishman et al. [35] introduced a robust moving least squares technique for fitting a piecewise smooth surface from a point set. The main tool that they use is a new robust statistics method for outlier detection: the *forward search *algorithm, which has a significant advantage in detecting outliers over commonly used backward methods. Unlike most existing backward methods, which depend on the initial estimating for the entire point set, the forward search starts from a small set of robustly chosen samples of the data that excludes outliers. Then the forward method moves forward through the data for adding observations to the subset while monitoring certain statistical estimates. Our work presented in this paper is in the same spirit and applies the forward search to structural superposition of flexible proteins. The main difference between our algorithm and the existing superposition methods is to replace "least squares" by "least median of squares" by combining the forward search such that the improved superposition algorithm is more robust for large displacements. Our method can be considered an alternative tool for structural superposition as a complement of other tools like sieve-fit, fit-all [18-20], and the wRMSD fit [2].

## Results

We have implemented the technique presented in the previous section and tested it on a number of proteins with known conformational changes. The algorithm described above is implemented in C++. In this paper, the execution time is given in seconds on a Pentium IV 1.70 GHz processor with 512M RAM excluding the time of loading proteins. For simplicity, our code and our examples in this article use only two conformations of each protein, but this algorithm could be applied into any program that iteratively superposition ensembles of structures.

**Figure 1:**
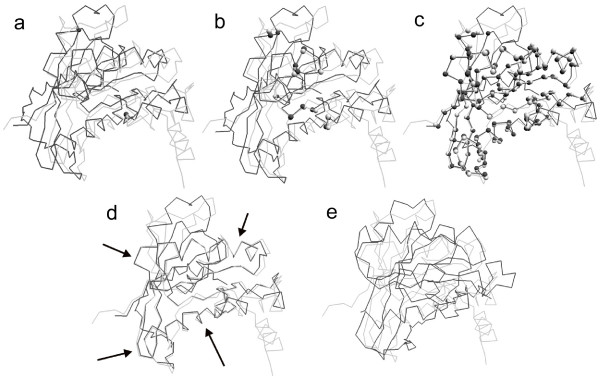
**The illustration of the LMS fit between two conformations (RAN): 1byu (light gray) and 1rrp (dark gray)**. First, an initial subset is selected using the LMS algorithm, as shown in (a). Next, we iteratively add one pair of points with the smallest residual and refit two conformations to the updated subset using the standard RMSD fit. The subset at 10th iteration is shown in (b). The final subset using the forward search is shown in (c). The remaining points are regarded as outliers that are not used for computation of the final superposition. The superposition results using the LMS and RMSD fit are given in (d) and (e), respectively. Arrows denote regions with improved fit.

Fig. 1 shows the procedure of the LMS fit between two conformations based on the forward search algorithm. First, an initial subset (two point pairs in Fig. 1(a)) is selected using the LMS algorithm. Next, we iteratively add one pair of points with the smallest residual and refit two conformations to the updated subset using the standard RMSD fit. The subset at 10th iteration is shown in Fig. 1(b). If the error is larger than a predefined threshold, the iteration procedure is terminated. The final subset is shown in Fig. 1(c). The remaining points are regarded as outliers that are not used for computation of the final fit. The superposition results using the LMS and RMSD fit are given in Fig. 1(d) and Fig. 1(e), respectively. Arrows denote regions with improved fit.

### Protein data set

We have chosen to test our method on protein systems found in the Database of Macromolecular Movements (MolMovDB) [40]. MolMovDB presents a diverse set of proteins that display large conformational changes in protein and other macromolecules, which can be found at: . The corresponding experimental structures are downloaded from the Protein Data Bank (PDB) [41], and the first chain of each structure is used as the reference structure for superposition. PyMOL is used for various visualization purposes and the creation of figures for this article [42].

Our code currently implements our method using *C*_*α *_coordinates of two protein conformations (it is straightforward to use all backbone atoms). Our preprocessing removes any inappropriate residues including duplicate residues, disordered residues, or heterogroups from the respective PDB file. We first apply the LMS fit to several protein systems in MolMovDB. Table 1 lists the names of test systems and gives the superposition results for each protein system in the final LMS fit, where "Protein system" is the name of the test system, "PDB1" and "PDB2" are PDB codes of two conformations fitted, "RMSD" is the standard RMSD distance for the entire atom sets using the RMSD fit, "#Res" is the number of atom pairs after removing the inappropriate residues, "#Subset" is the number of atom pairs in the final subset, "Core%" is the proportion of the core region (i.e. the final subset) that belongs to the original point set (see Eq. (4)), and "Time(s)" is the time of computing the LMS fit. The proteins are chosen based on their interest to the community, variation in size, and range of conformational changes. When the structures between two conformations are very similar (e.g. RAN and ER*α*), there is usually a high "Core%". In contrast, the lower the similarity, the smaller the value of "Core%" (e.g. Calmodulin and Myosin). The presented algorithm is also fast. For instance, it performs a structural superposition for a pair of conformations with 700 amino acids in about half a second.

**Table 1:** Superposition results of protein systems with conformational changes using the LMS fit

Protein system	PDB1	PDB2	RMSD	#Res^1^	#Subset	Core%^2^	Time(s)
ER*α*	3erd	3ert	4.9	244	203	83.2%	0.28
RAN	1byu	1rrp	14.4	200	141	70.5%	0.19
Myosin	1b7t	1dfk	13.0	720	403	56.0%	0.58
Calmodulin	1cll	1ctr	14.7	138	72	52.2%	0.09
Topo II	1bgw	1bjt	18.4	665	389	58.5%	0.55
Pneumolysin	2bk1	2bk2	21.8	435	139	32.0%	0.39

^1 ^"#Res" is the number of atom pairs used for superposition by removing any inappropriate residues.

^2 ^"Core%" is the proportion of the core region that belongs to the original point set.

The superposition procedure first requires one to create a list of corresponding atom pairs; and then performs a LMS fit to bring the two proteins into proximity. Note that the LMS fit is not a tool for structure-based sequence alignment, which is a separate bioinformatics challenge [8,43]. Thus, like other structural superposition methods [2,8], the LMS fit requires a prior one-to-one mapping among the atoms/residues in the structures under consideration. Our method can be applied to align two homologous structures with different residues by incorporating some initial sequence or structural comparison to create the corresponding atom pairs.

### Parameters

The LMS fit algorithm presented in this paper involves two parameters: the maximal residual *r*_max _(default is 2Å) and the minimal iteration number MIN_ITERS (default is [*N*/2.0]). Here, MIN_ITERS is usually chosen as a predefined integer to ensure that the number of atoms on core regions is more than 50% of entire atoms. In this section, we start by investigating the effect to the maximal residual *r*_max_. The threshold *r*_max _controls the final subsets fitted. In order to investigate only the effect of *r*_max_, we first ignore the another termination condition that the iteration number should be larger than the minimal iteration number MIN_ITERS.

#### The maximal residual

**Figure 2:**
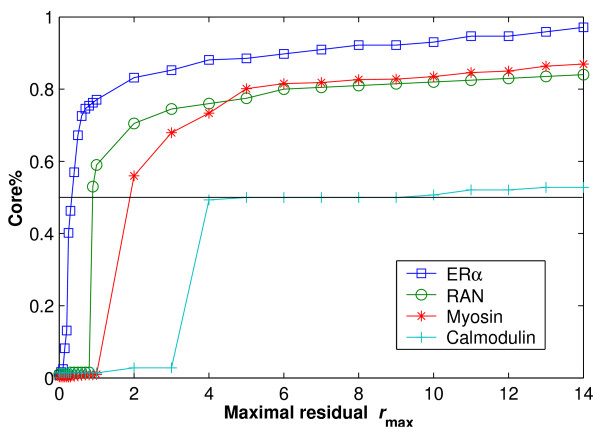
**The behavior of the core region Core% with different maximal residuals *r*_max _for four protein systems, where the horizontal line indicates where Core% = 0.5 is reached**.

Fig. 2 shows the value of Core% with respect to the various *r*_max _for four protein systems: ER*α*, RAN, Myosin and Calmodulin, which are referred to in Table 1. We vary the threshold of the maximal residual, using *r*_max _from 0 to 14Å, to determine its effect on the LMS fit. The value of Core% increases with *r*_max _until to 100% reached for the entire atom pairs. This reason is that the LMS fit adds the atom pair with the minimal residual into the current subset at each iteration until all atom pairs are exhausted. When the structures are very similar, a small *r*_max _can obtain a "tighter fit" of the rigid core with a high value of Core%. For instance, *r*_max _= 1.0Å can get a value of Core% close to 80% for the ER*α *structure. In contrast, when the structures are dissimilar on large regions, a large *r*_max _is required. Note that *r*_max _more than 4.0Å can only get about Core% = 50% for the Calmodulin structure. Therefore, we found that it is not sufficient to superimpose all protein systems with high and low similarity if we only use a fixed *r*_max_. To overcome this problem, we suggest that the maximal residual *r*_max _and the minimal iteration number MIN_ITERS are combined for controlling the termination conditions. For protein systems with high similarity, *r*_max _= 2.0Å usually is enough for obtain an appropriate subset. If when *r*_max _= 2.0Å is not sufficient for protein systems with low similarity, MIN_ITERS can assure the number of the fitted subset is more than 50% of entire atoms. We found that the combination of *r*_max _and MIN_ITERS with defaults can lead to fast convergence and little computation time for most protein systems in MolMovDB. In all results shown in this paper, we use *r*_max _= 2Å and MIN_ITERS = [*N*/2.0] for obtaining both small errors and little computation time.

### Comparison of results

In this section, we first compare the visualization results of structural superposition for some conformations. Then we present a strategy, called *residual histogram*, for quantifying the superpositions.

#### Visualization comparison of superposition

In this section, we compare the performance of our algorithm with three superposition techniques: the RMSD fit, sieve-fit, and the wRMSD fit [2]. The sieve-fit source code can be found on the Gerstein Lab website , where we use the default parameters (the maximal iteration number is 500 and the distance threshold is 0.5). The wRMSD source code is available on the Carlson Lab website . The Gaussian weight of wRMSD is computed by $w_{n}=e^{-{\left(d_{n}\right)}^{2}/c}$, where *c *is a scaling factor and *d*_*n *_is the distance between atom *n *in each protein conformation. In the old version of wRMSD fit, *c *is set to 2Å for similar structures; *c *is set to 5Å for non-similar structures. In structures with radical changes, the scaling factor may be as high as the initial RMSD between the structures. There are two programs (the global and local wRMSD fit) available. The local wRMSD is the recommended algorithm on proteins with extreme structural changes. Recently, Damm and Carlson updated the global wRMSD code that set the scaling factor to the standard RMSD value. The wRMSD fit can produce good structural superposition of two conformations with small and large displacements. The LMS and wRMSD fit achieves the similar results.

**Figure 3:**
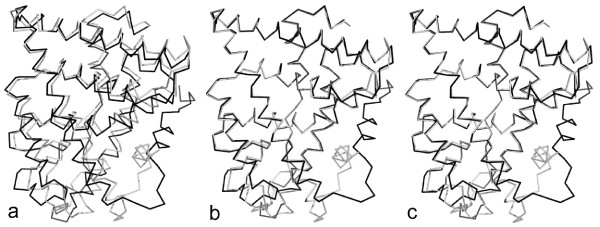
**Superposition comparison for the ER*α *structures: 3erd (light gray) and 3ert (dark gray)**. (a) The RMSD superposition. (b) The LMS superposition, where the maximal residual *r*_max _of 2Å is used in our method. (c) The wRMSD superposition. For small displacements, our method can get the almost consistent result with wRMSD.

**Example 1**. The ER*α *structures (3erd and 3ert) are tested using the RMSD, LMS and wRMSD fit, where there are some small structural changes between 3erd and 3ert. Fig. 3 shows the results of superposition for ER*α *using three methods. In the final RMSD fit (Fig. 3(a)), only 39 of 244 atom pairs common to both structures are within 1Å. Contrastively, the final LMS fit (Fig. 3(b)) has 188 atom pairs within 1Å, and the RMSD distance between two core regions (203 atom pairs) is 0.49Å. In addition, the final wRMSD fit (Fig. 3(c)) has also 188 atom pairs within 1Å. In Figs. 3(b) and 3(c), we observe that the fit results of LMS and wRMSD are very similar. When the change between two conformations is slight, the result of superposition using the LMS fit is approximately equal to one using the wRMSD fit [2]. Both LMS and wRMSD are able to highlight the similarity of the rigid core regions better than RMSD.

**Figure 4:**
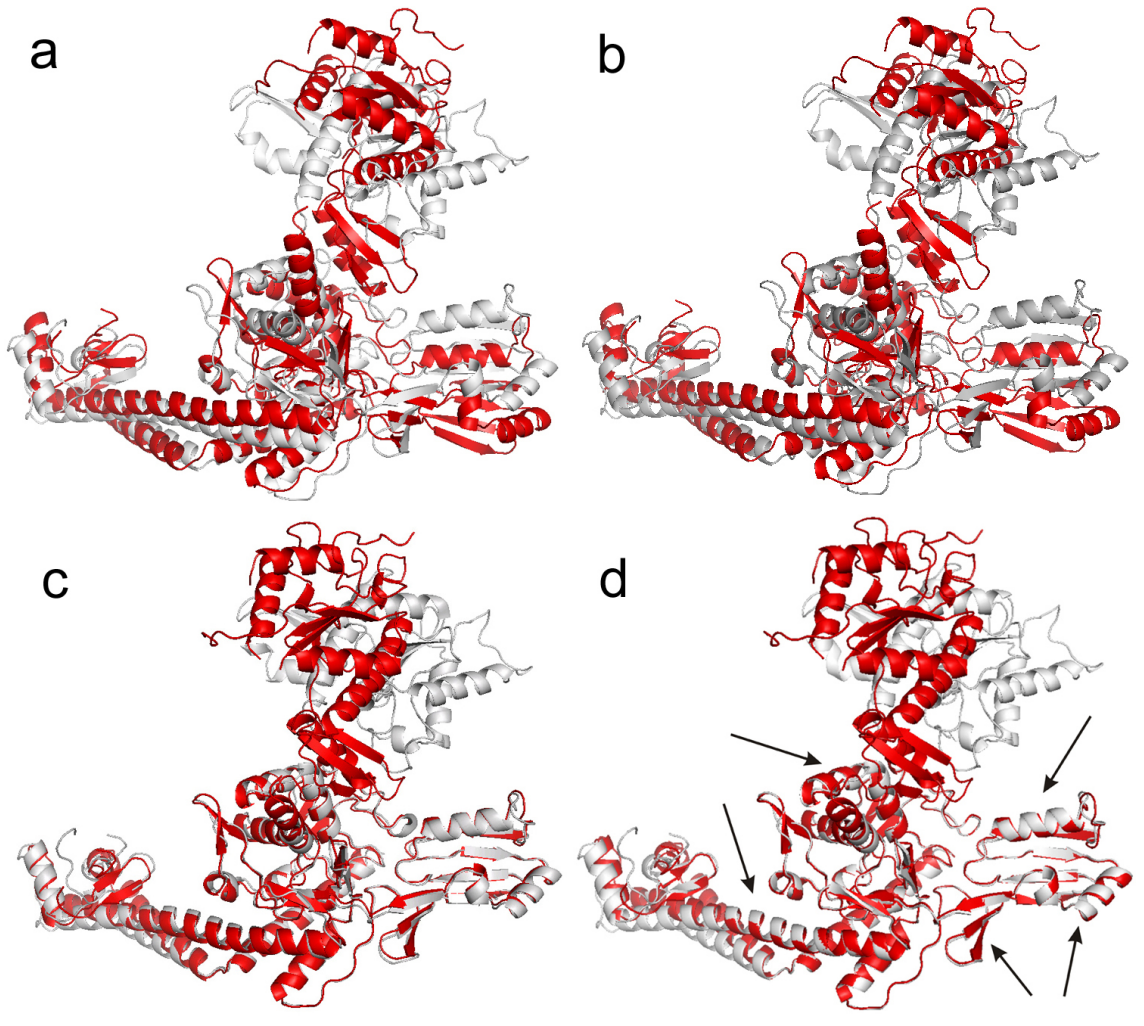
**Superposition comparison for the Topo II structures: 1bgw (gray) and 1bjt (red)**. (a) The RMSD superposition. (b) The sieve-fit superposition. (c) The global wRMSD superposition (*c *= 18Å). (d) The LMS superposition, where the maximal residual *r*_max _of 2Å is used in our method. For large displacements, both RMSD and sieve-fit can not catch the similarity of two conformational structures. In contrast, the LMS fit can catch the similarity, where the LMS result is similar to one using wRMSD with the default scaling factor. Arrows denote the improved regions with the LMS fit.

**Example 2**. The Topo II structures (1bgw and 1bjt) are tested using four methods, where there are some large structural changes between 1bgw and 1bjt. Fig. 4 shows the results of superposition for Topo II. Different crystal forms exhibit significant changes in overall architecture of Topo II, including an extremely large (170 degrees) domain rotation [44]. The changes between two conformations are too large such that the standard RMSD fit misses the structural similarity, as shown in Fig. 4(a). The final superpositions using the standard RMSD and the sieve-fit have 26 and 18 atom pairs within 2Å, respectively. The final LMS fit has 381 atom pairs within 2Å, and the RMSD distance between two core regions (389 atom pairs) is 0.85Å. Arrows in Fig. 4(d) highlight the improvement in fitting the rigid core of Topo II. The LMS fit can catch the structural similarity and our result is similar to one using the wRMSD fit with the default *c*, as shown Fig. 4(c).

**Figure 5:**
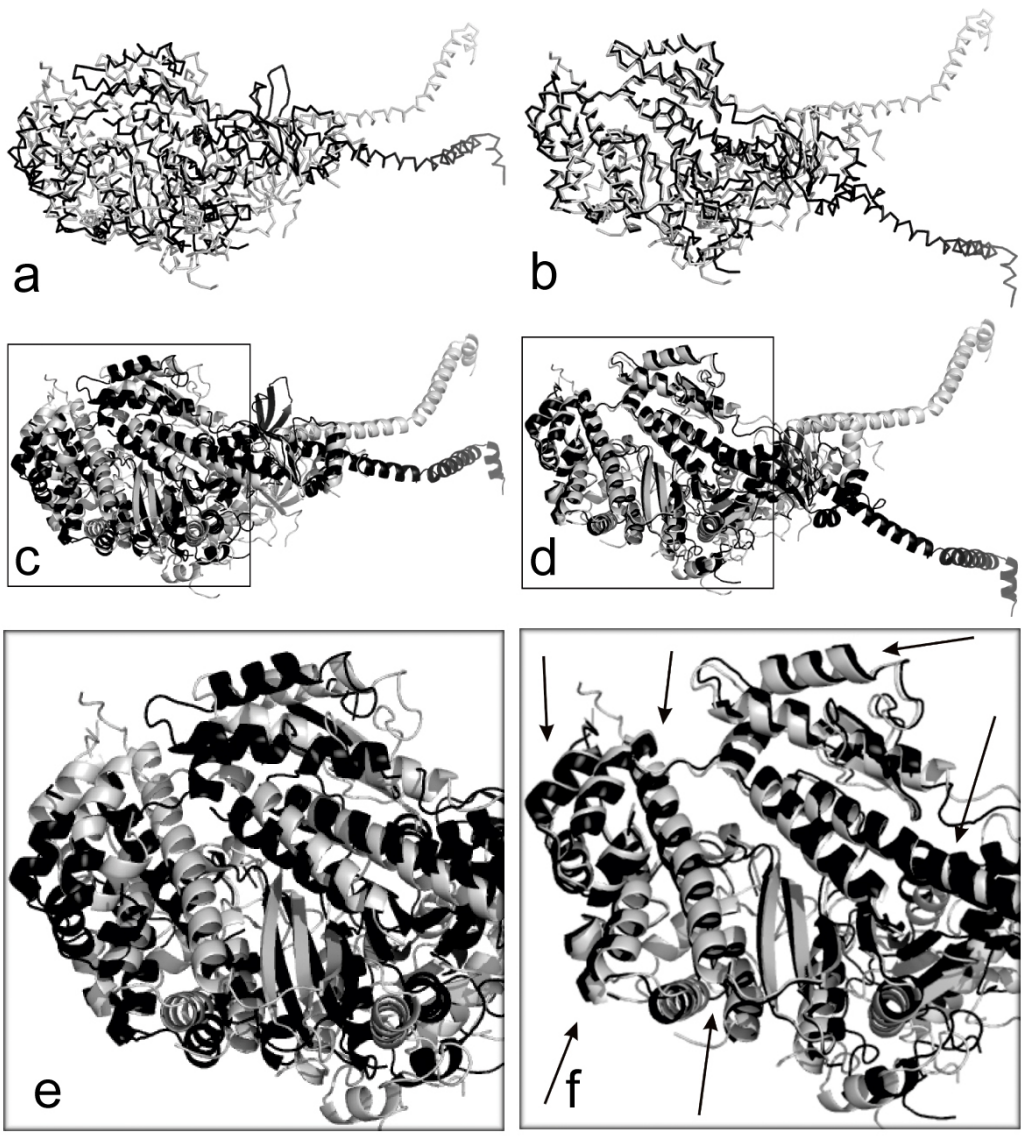
**Superposition comparison for the Myosin structures: 1b7t (light gray) and 1dfk (dark gray)**. (a) The RMSD superposition. (b) The LMS superposition. (c) and (d) show the secondary structures corresponding to (a) and (b). (e) and (f) are the magnified views of (c) and (d), respectively. Arrows denote regions with improved fit.

**Figure 6:**
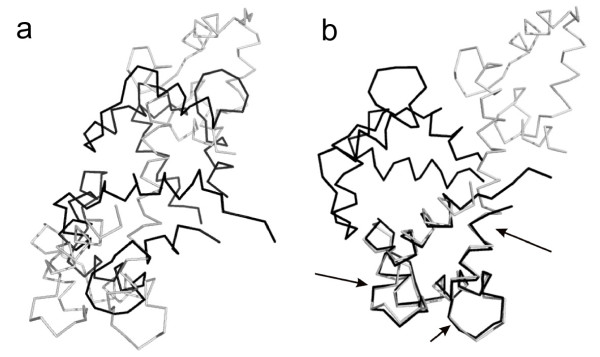
**Superposition comparison for the Calmodulin structures: 1cll (light gray) and 1ctr (dark gray)**. (a) The RMSD superposition. (b) The LMS superposition. Arrows denote regions with improved fit.

**Example 3**. Figs. 1, 5 and 6 demonstrate the superposition results for three protein systems: RAN, Myosin and Calmodulin, which have large conformational displacements. In these figures, arrows highlight regions with improved fit using our method. The LMS fit takes about 0.19s, 0.58s and 0.09s, respectively. In the first protein system, the RAN structures (1byu and 1rrp) have large conformational changes, and the movement occurs in two switch regions. For the RAN structure, the final RMSD fit only captures 2 of the 200 atom pairs within 1Å; the final LMS fit keeps 116 atom pairs within 1Å. In the second protein system, the Myosin structures (1b7t and 1dfk) have much larger conformational changes, where the largest movements produced are more than 50Å. For the Myosin structures, the LMS fit contains 402 of the 720 atom pairs within 2Å, but there are only 30 atom pairs within this range when using the RMSD fit. In the third protein system, Calmodulin is a ubiquitous, calcium-binding protein that can bind to and regulate a multitude of different protein targets. We superimpose two conformational structures (1cll and 1ctr) of Calmodulin, where this hinge motion involves a long helix splitting into two helices and the angle between the axes of the two helical segments is about 100 degrees. Furthermore, as there is an additional twist around the helix axes, the total rotation of one domain relative to the other is upwards of 150 degrees. The final RMSD fit can not detect any atom pairs within 2Å; contrastively, the final LMS fit has 69 of the 138 atom pairs within 2Å.

**Figure 7:**
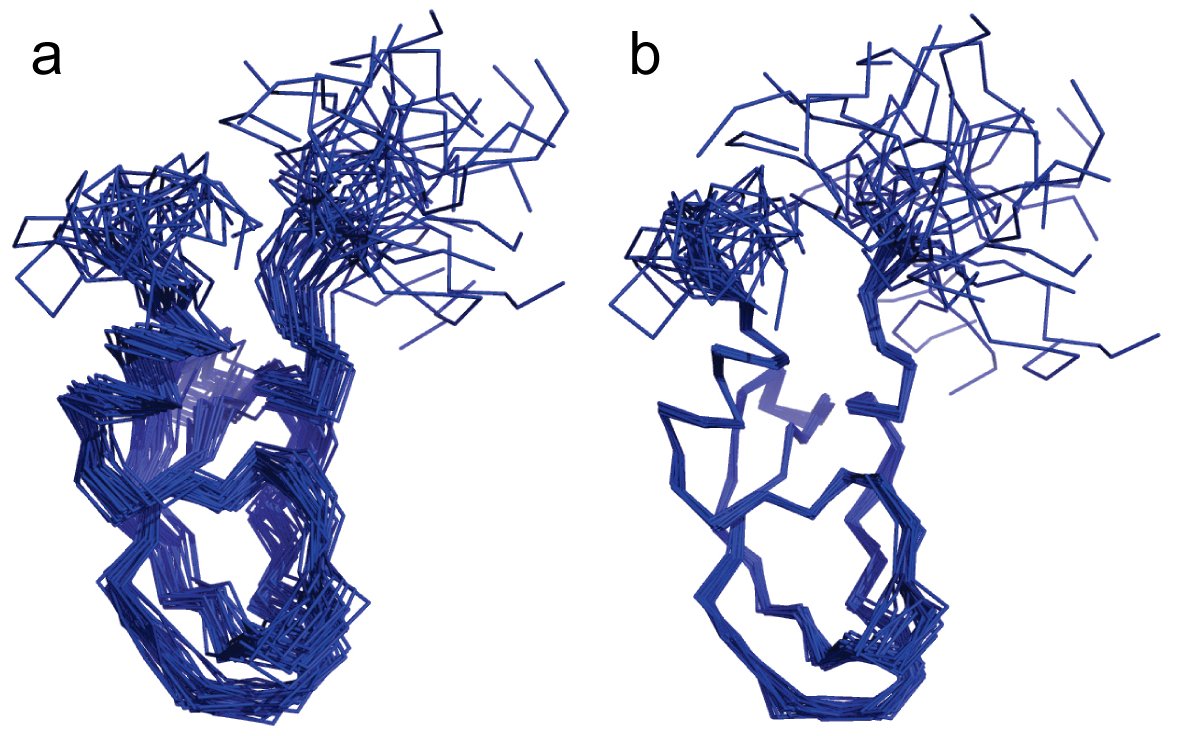
**Superposition comparison for 30 NMR models of the second Kunitz domain of Tissue Factor Pathway Inhibitor **(PDB ID: 1adz). Here all conformations are superimposed with a reference structure (the first model) using the RMSD and LMS fit. (a) The RMSD superposition. (b) The LMS superposition.

**Example 4**. Finally, we compare a conventional LS superposition and the LMS superposition for 30 NMR models of the second Kunitz domain of Tissue Factor Pathway Inhibitor (PDB ID: 1adz), as shown in Fig. 7. Here all conformations are superimposed with a reference structure (the first model) using the RMSD and LMS fit. In Fig. 7(a), the RMSD superposition provides misleading and inaccurate results; the LMS superposition in Fig. 7(b) can catch the similarity of multiple conformational structures, contrastively. This example in Fig. 7(a) is also used for demonstrating advantages of maximum likelihood superposition when assuming a Gaussian distribution of the whole structures in the analysis by Theobald et al. [8,16]. Our LMS superposition obtains the almost consistent result with maximum likelihood superposition for multiple structures.

#### Residual histogram

**Figure 8:**
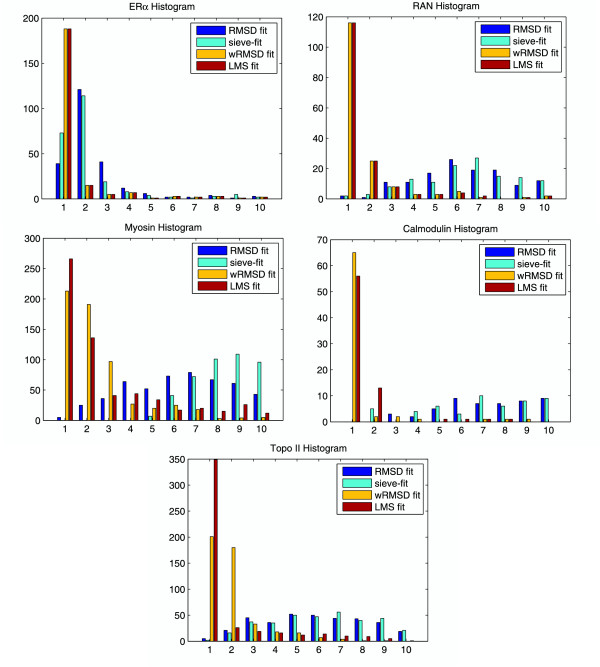
**Residual histograms of five protein systems (ER*α*, RAN, Myosin, Calmodulin, and Topo II) in the final superpositions**. Here a histogram is constructed by segmenting the distance from 0Å to 10Å into 10 equal sized ranges (each range is 1Å) and counting the number of atom pairs whose distance is within each ranges. The horizontal axis of the histogram is labeled with the range (Å) of residual of atom pairs, and the vertical axis of the histogram is the number of atom pairs whose residuals are within the corresponding range. Note that the number of counts for the LMS and wRMSD superpositions within the range of 0 – 1Å is far larger than one for the RMSD and sieve-fit superpositions.

In this section, we use a residual histogram for demonstrating the residual distribution of atom pairs for the final LMS and RMSD fit. Fig. 8 shows the residual histograms of five protein systems (ER*α*, RAN, Myosin, Calmodulin and Topo II) described above for the final RMSD, sieve-fit, the global wRMSD, and LMS superposition. Here a residual histogram is constructed by segmenting the length 0 – 10Å into equal sized ranges (1Å) and counting the number of atom pairs whose residuals are within each range. The horizontal axis of the histogram denotes the ranges segmented and the vertical axis is the number of counts. For example, in ER*α *Histogram in Fig. 8, the first "LMS fit" bar on left denotes that there are 188 atom pairs whose residuals are within the range of 0 – 1Å for the ER*α *structures in the final LMS fit; and the second "LMS fit" bar on left means there are 15 atom pairs whose residuals are within the range of 1 – 2Å. In contrast, the first "RMSD fit" bar on left denotes that there are 39 atom pairs within the range of 0 – 1Å in the final RMSD fit.

The LMS fit tends to fit the rigid core of two conformations and ignore the effect of the flexible regions. Therefore, the atom pairs with little movement between two conformations will have a small residual (usually within 0 – 1Å) in the LMS fit. In contrast, these atom pairs are effected by the flexible regions in the RMSD fit. Although the RMSD fit minimizes the sum of distance of entire atom pairs, it can not guarantee the small residuals to the majority of atom pairs. In fact, the RMSD fit is only the minimization in the sense of average. In the final RMSD fit, each atom pair shares both little movement on the core regions and large movement on the flexible between two conformations. In the examples in Fig. 8, we observe that the number of counts for the LMS fit within the range of 0 – 1Å is far larger than one for the RMSD fit. In special, Calmodulin Histogram in Fig. 8 shows that no atom pair is within two ranges of 0 – 1Å and 1 – 2Å in the final RMSD fit for two conformations of Calmodulin, whereas 69 of the 138 atom pairs are within the two ranges in the final LMS fit. In contrast, the wRMSD fit achieves similar results with the LMS fit (especially within 0 – 2Å), while there are few atom pairs within the range of 0 – 2Å in the final sieve-fit.

**Figure 9:**
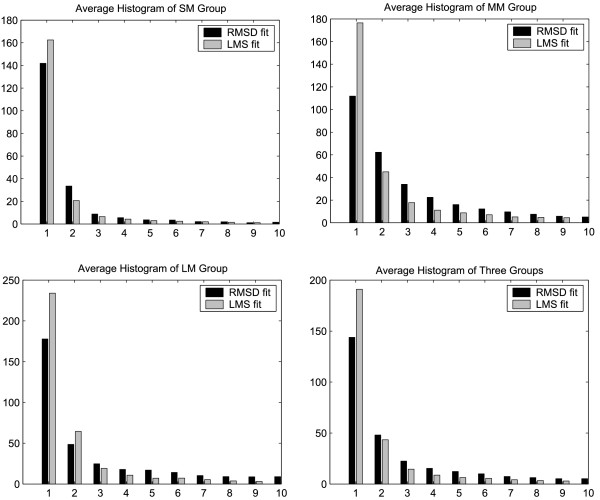
**The average residual histograms for a collection of known protein systems with conformational changes in MolMovDB**.

Finally, to obtain a broader overview we apply the LMS fit to a collection of known protein systems with conformational changes in MolMovDB (as of October 2007). The conformational database is classified by the size of the mobile regions as three groups: 1) motions of fragments smaller than domains, 2) domain motions, and 3) larger movements than domain movements involving the motion of subunits. We simply call the three groups: SM (small movement), MM (medium movement) and LM (large movement). There are 56, 123 and 22 protein systems that are available in the three groups, respectively. For these examples shown in Table 1, ER*α *is selected from the SM group, Topo II is selected from the LM group, and the other protein systems are selected from the MM group except Pneumolysin. Especially, the motions of RAN and Calmodulin is predominantly hinge type and Topo II has complex protein motion. All protein systems have at least one pair of conformations, and animations of the conformational transition are available for most protein systems. To avoid bias from large families with multiple conformations, we retained only one pair of conformations per protein system, leading to 201 pairs of conformational structures. The same parameters (*r*_max _= 2Å and MIN_ITERS = [*N*/2.0]) are used in all the calculations. Fig. 9 shows the average residual histograms for protein systems in SM, MM, LM, and three groups in the final superpositions using the RMSD and LMS fit. The final LMS fit has the average of 163, 177, and 234 atom pairs within 0 – 1Å for the SM group, the MM group, and the LM group, respectively; whereas the final RMSD fit only has the average of 141, 111 and 177 atom pairs within the this range. The average of 192 atom pairs for three groups is within 0 – 1Å in the final LMS fit; the average of 143 atom pairs is within this range in the final RMSD fit. In the final LMS fit for three groups, the average value of Core% is 79.7%, and the average RMSD distance in the core regions is 1.1Å.

### Multiple level superposition

It was previously shown that there is generally not a unique solution for the structural fit between two prteins [2,15]. If two different conformations each consists of multiple rigid domains, our LMS fit algorithm will get the subset in the biggest rigid domain for computing the superposition. An extension version of our algorithm can also be extended to multiple level superposition between two protein conformations with several rigid domains. Given two conformations **X **and **Y **with multiple several rigid domains, we present an iterative algorithm for determining multiple level superposition of **X **and **Y **as follows.

1. First, we compute the core regions *Q*_x _and *Q*_y _of two conformations **X **and **Y **using the LMS fit algorithm and identify the rest of the data as outliers.

2. Next, we remove the core regions *Q*_x _and *Q*_y _from **X **and **Y**, and update two conformations as **X **= **X **- *Q*_x _and **Y **= **X **- *Q*_y_, respectively. Then we recompute the LMS fit between the updated **X **and **Y**.

3. The above Steps 1 and 2 are repeated until the superposition level defined by users is reached, where the superposition level denotes which level rigid domain is finally superimposed. The final centers and rotation matrix are computed by the final level rigid domain.

**Figure 10:**
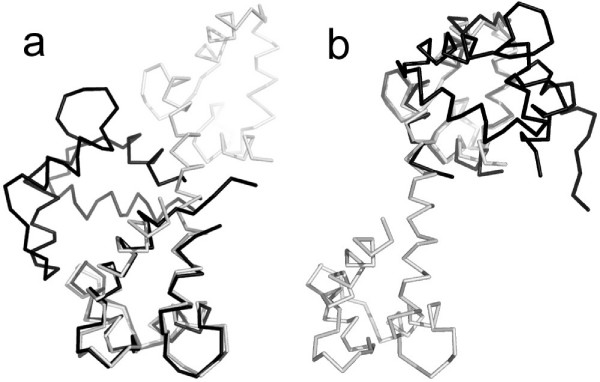
**Multiple level superposition for the Calmodulin structures: 1cll (light gray) and 1ctr (dark gray)**. (a) The first level superposition (Core% = 51.4%). (b) The second level superposition (Core% = 46.4%). Note that our method can capture two rigid domains in two level superposition, respectively.

**Figure 11:**
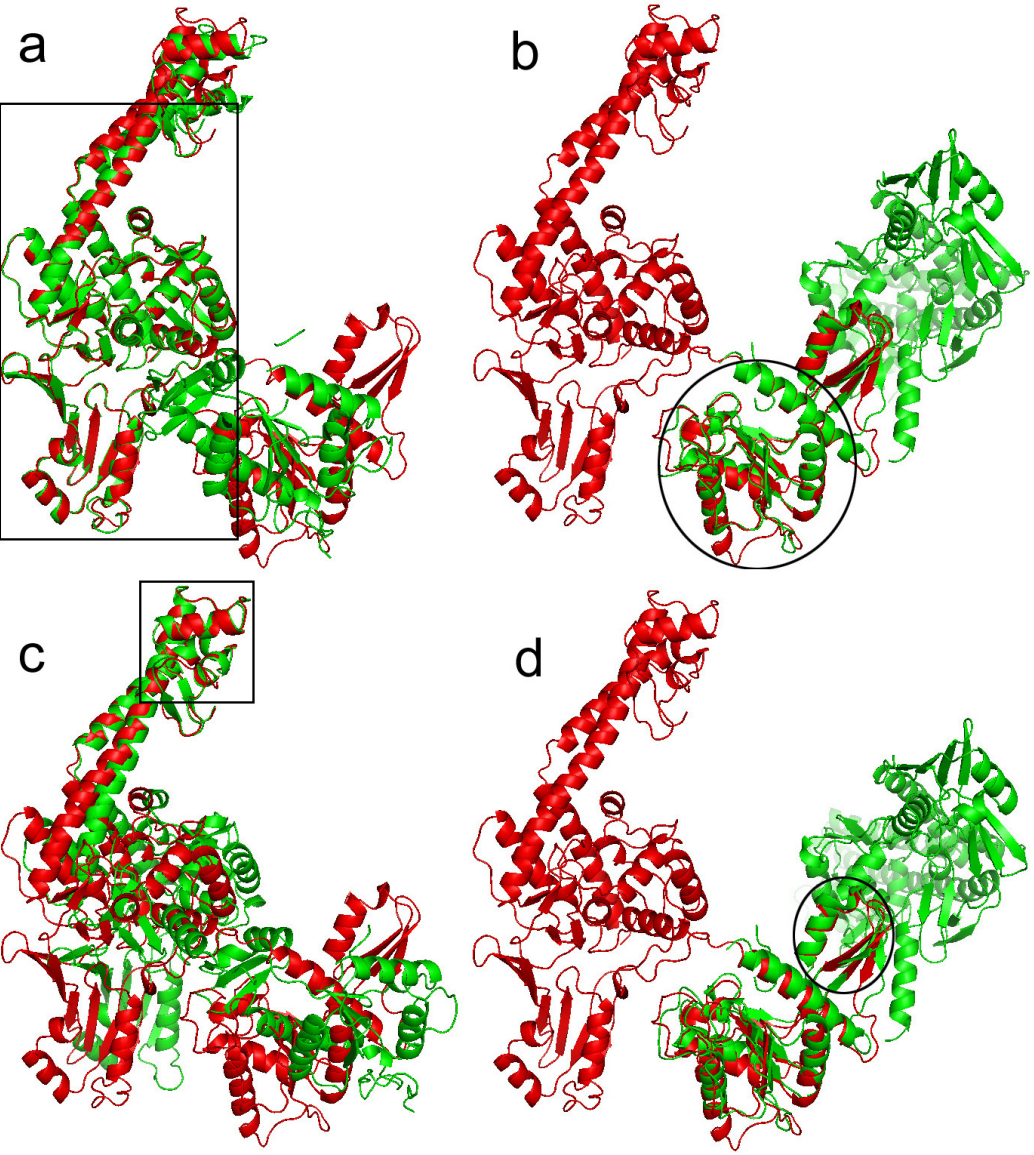
**Multiple level superposition for Topo II: 1bgw (red) and 1bjt (green)**. (a) Level 1 (Core% = 56.4%). (b) Level 2 (Core% = 22.1%). (c) Level 3 (Core% = 11.7%). (d) Level 4 (Core% = 5.1%). Note that our method can capture different rigid domains in multiple level superposition, where the superimposed rigid domains are highlighted in the selected regions with the solid line boundary.

**Figure 12:**
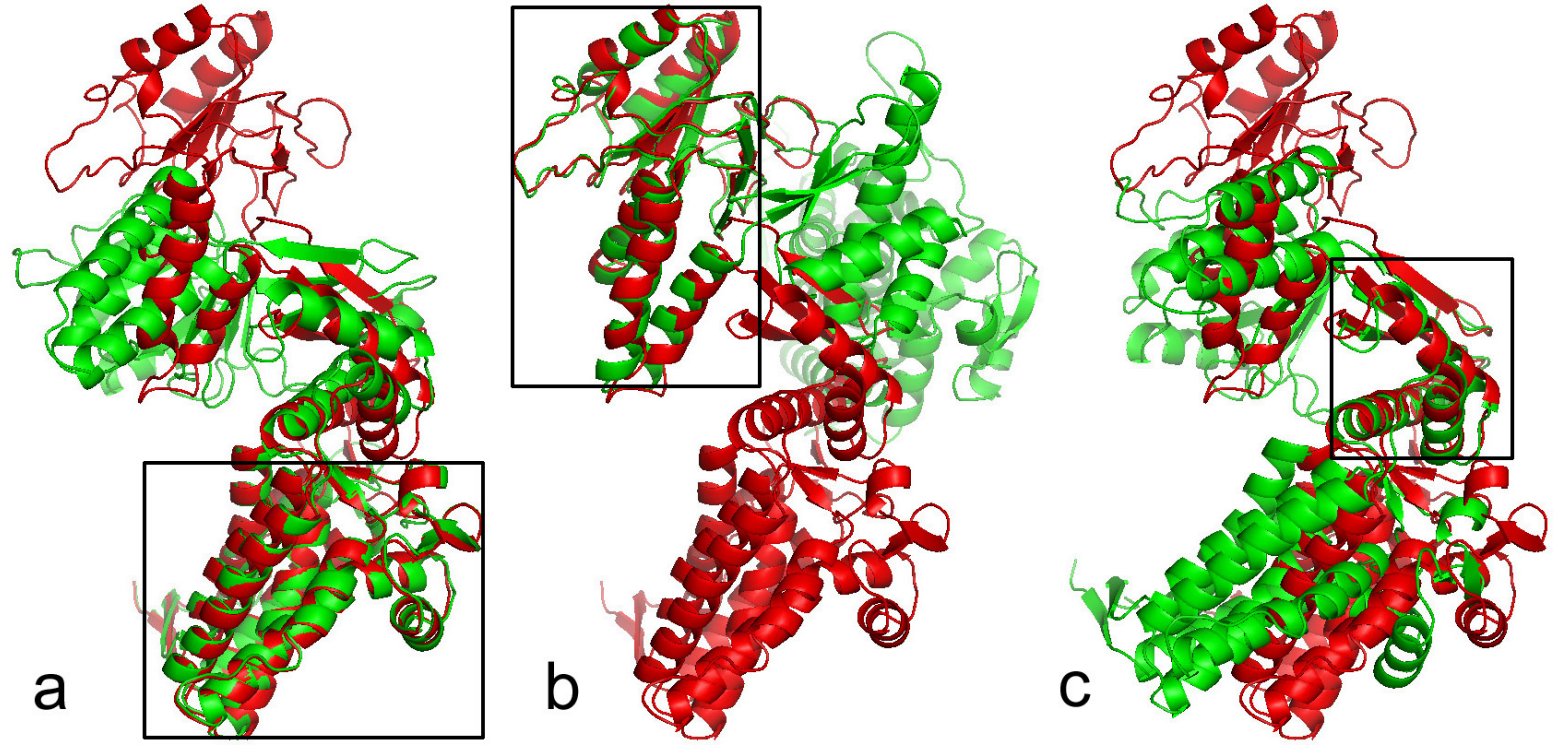
**Multiple level superposition for GroEL: 1aon (red) and 1kp8 (green)**. (a) Level 1 (Core% = 47.5%). (b) Level 2 (Core% = 30.5%). (c) Level 3 (Core% = 11.6%). Note that our method can capture three different rigid domains with three levels, where the superimposed rigid domains are highlighted in the selected regions with the solid line boundary.

Several examples are shown in Figs. 10, 11, 12 for demonstrating the multiple level superposition algorithm. Fig. 10 illustrates two level superposition for the Calmodulin structures: 1cll (light gray) and 1ctr (dark gray). The first level superposition has one common big rigid domain with Core% = 51.4% in Fig. 10(a), and the second level superposition has one common small rigid domain with Core% = 46.4% in Fig. 10(b). Fig. 11 gives four level superposition for Topo II: 1bgw (red) and 1bjt (green). Fig. 12 gives three level superposition for GroEL: 1aon (red) and 1kp8 (green). Note that our method can capture several different rigid domains with multiple levels, where the superimposed rigid domains are highlighted in the selected regions with the solid line boundary.

The multiple level superposition algorithm is actually the extension of the LMS fit. This algorithm can be performed through a parameter 'level' without specifying and choosing any residues. The local wRMSD fit can finish a similar function as multiple level superposition by sampling some subsets of the protein for changing the initial RMSD fit in advance [2].

## Discussion

In this section, we will discuss median measurement changing and comparison of similarity scores.

### Changing median measurement

**Figure 13:**
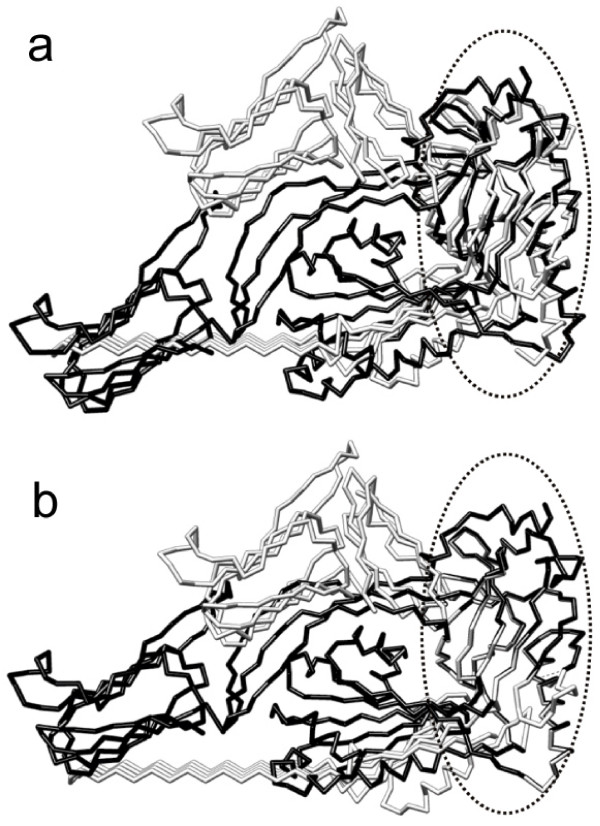
**If the number of atom pairs on the flexible regions is larger than one on the core region, the LMS fit based on the minimal median measurement can not get good superposition**. (a) The LMS fit for the Pneumolysin system: 2bk1 (light gray) and 2bk2 (dark gray), using the minimal median measurement. (b) The improved superposition through changing the parameter of the LMS fit, i.e. replacing the median (50%) by the first quartile (25%). The superposition differences are highlighted in the ellipse regions with the dashed boundary.

If the flexible regions between two conformations are too large such that the rigid core region contains less 50% atoms of the entire atom sets of protein, we do not see good superposition using the LMS fit based on the minimal median assumption. Fig. 13 demonstrates this issue using the Pneumolysin structures (2bk1 and 2bk2 from CryoEM) [45]. In Fig. 13(a), when the LMS fit based on the minimal median measurement is applied for two conformations, we do not see the good superposition. The main reason is that the rigid core region only contains about 30% atoms of the entire atom sets of protein. The special case is not usual, and there are few protein systems like Pneumolysin in MolMovDB.

For this case that the flexible regions contain more atoms than the core region, we can simply change the "median" parameter in the LMS fit for improving the superposition. At the phase of initial subset selection, the original LMS fit uses the random sampling algorithm for selecting *k *initial point pairs with a small value of *k*. At each iteration, 1) *k *point pairs are first selected between two point sets at random; 2) then the median of the residuals of the remaining point pairs is computed; 3) finally, *k *point pairs with the minimal median are selected as the initial subset for the forward search. Instead of the minimal median measurement, we may use the *m*th smallest value from the residuals of the remaining point pairs for improving the initial point pairs. In Fig. 13(b), we use the first quartile (25%) instead of the median (50%) for cutting largest 75% outliers. The first quartile actually assumes that the flexible regions contain up to 75% atoms of the entire atom sets of protein. The superposition difference is highlighted in the ellipse regions with the dashed boundary.

### Comparison of similarity scores

One application of comparing two conformations of the same protein sequence is to evaluate a predicted protein structure against its experimentally determined target. We examine one system Target 179 (PDB ID: 1IY9) in the CASP5 competition [46] for comparing our similarity score with three ones (GDT_TS, TM-score and wRMSD's scores). The GDT_TS values can be obtained from the CASP5 website , and the TM-score [47] can be computed from TM-score online . The specific target has been discussed in Damm and Carlson's work [2], and the wRMSD's scores (%wSUM and %wSUM_ALL) discussed here are directly cited from their paper. Similar to their strategy, we provide a Core% score based on the fit of the coordinates in the prediction (*N *in Eq. (4) equals the number of atoms in the prediction) and a Core_All%, which corrects for any omitted coordinates (*N *in Eq. (4) equals the number of atoms in the target). If a prediction provides all *C*_*α *_coordinates, Core% and Core_All% are equal. GDT_TS (Global Distance Test_Total Score) evaluates two structures based on the RMSD fit of a subset of atoms in an iterative weighted evaluation, and TM-score is an extension of GDT. %wSUM and %wSUM_ALL scores are the average of weight values in the final wRMSD superposition.

Damm and Carlson randomly selected some good, exceptional and poor submissions from Target 179's groups. We use the same data. Since some poor submissions are included in the groups, we choose the first quartile (25%) as the measurement parameter instead of the median (50%). Table 2 shows that the rankings provided by Core_All%. Core_All% scores match %wSUM_ALL and GDT_TS with the exception of groups 32 and 270. Damm and Carlson have analyzed that the cause for 32's poor GDT_TS rank may be a simple typographical or data processing error. In contrast, TM-score gives a top ranking for 32 group liking Core_All% (the top one in %wSUM_ALL and second one in %wSUM). Group 270 has also the different ranking among %wSUM_ALL and TM-score. By superposition, we found that group 270 is a good predictions and it looks very similar to the target. The small ranking difference between three methods may be reason of the weight values. The LMS scores (Core% and Core_All%) can be considered an alternative and complementary similarity score for assessing the quality of protein conformations.

**Table 2:** Target 179 (PDB ID: 1IY9) LMS rankings compared to %wSUM, GDT_TS and TM-score values

Group	Core_All%	Core%	%wSUM_ALL	%wSUM	GDT_TS	TM-score
032	88.7	89.7	76.5	77.0	28.65	93.1
427	88.3	88.3	76.6	76.6	86.95	92.6
270	88.2	88.2	74.6	74.6	84.40	91.9
246	88.0	88.0	76.3	76.3	86.68	92.5
471	86.1	86.1	75.8	75.8	85.77	91.8
016	82.5	62.5	64.0	64.0	77.47	90.0
529	72.6	85.4	63.8	75.1	72.08	90.3
291	27.4	42.6	24.0	37.4	34.12	63.8
400	20.4	35.2	18.9	32.6	29.11	60.2

## Conclusion

We have presented a novel technique of structural superposition for flexible proteins. The method is based on least median of squares (LMS) for guiding the classical RMSD fit. The forward search technique is used for approximating the LMS optimization. Using the method, we can automatically identify portions of proteins as the rigid core regions and flexible regions. The method does not require a prior knowledge of the flexible regions. Our fit tool has produced successful superposition when applied to proteins in MolMovDB for which two conformations are known. We also show that the LMS fit can be extended to multiple level superposition between two conformations with several rigid domains. This method can easily be incorporated into many RMSD overlay calculations. Note that LMS can not be a substitute for LS in some cases, such as the applications of LS to molecular dynamics (MD).

## Methods

### Least median of squares (LMS) fit

To overcome the lack of robustness using least squares fit in Eq. (1), some robust methods might be used for improving the RMSD fit, such as making use of some weight functions for bounding the influence of outliers [2]. Most existing robust methods are *least sum of squares *(also named *least squares *or LS), which can not raise a high breakdown point [36].

In our case, we assume that two different conformations of the same protein consists of two parts: the rigid *core *regions with high structural stability and the remaining *flexible *regions, and there is no overlap between them. Atoms in the core regions barely move between the two conformations. Indeed, the goal of the above assumption is to distinguish two different conformations as the "good" and "bad" parts. The core regions are assumed to contain at least 50% points of the entire point set, so the remaining flexible regions have up to 50% points. In our work, we treat the flexible regions as outliers. Our motivation is to improve the least sum of squares in the RMSD fit using a fit method with a high breakdown point (up to 50%). The *least median of squares *(LMS) is a robust statistics method that estimates the parameters of the model by minimizing the median of the absolute *residuals*. In other words, LMS replaces the sum of least squares by a *median*. The breakdown point of LMS is as high as 50% [36]. The resulting estimator using LMS can resist the effect of nearly 50% of contamination in the input data, which is applicable to our case. Given a rotation matrix **U**, the absolute residual is defined as the distance between the rotation point ${x^{\prime }}_{i}$ = **Ux**_*i *_and the target point **y**_*i*_; for the *i*th point pair the residual is *r*_*i *_= ||${x^{\prime }}_{i}$ - **y**_*i*_||. Based on the given **U**, the median of absolute residuals between two point sets is defined as:

(2) $$\begin{array}{cc} D_{median}=\underset{i}{\text{median}}\left\|Ux_{i}-y_{i}\right\|, & 1\leq i\leq N. \end{array}$$

In this paper, our goal is to search a best rotation matrix **U **that minimizes the median *D*_*median *_of the residuals as follows:

(3) $$\underset{U}{\min}\begin{array}{cc} \underset{i}{\text{median}}\left\|Ux_{i}-y_{i}\right\|, & 1\leq i\leq N, \end{array}$$

where **U **is the optimal rotation matrix that will be computed. Rousseeuw [36] has also pointed out there always exists a solution for LMS.

#### Random sampling algorithm for computing the LMS optimaztion

Eq. (3) can be solved using the following *random sampling *algorithm (i.e. RANSAC) [35,48]. First, *k *point pairs are randomly selected between two point sets, and the first rotation matrix is computed using the RMSD algorithm to the *k *point pairs. Next the median of the residuals of the remaining *N *- *k *point pairs is computed. The process is repeated *T *times to generate *T *candidate rotation matrices. The matrix with the minimal median is selected as the final rotation matrix **U**. A small value of *k *does not use all of the available points to the fit computation, while a larger value of *k *requires more iterations. If *k *is too large, the algorithm becomes sensitive to outliers, i.e. local displacements.

### The forward search

The forward search algorithm [39] is a new robust method that avoids the need to fix *k*. Recently, Fleishman et al. [35] have applied this technique to fit surfaces from point clouds in computer graphics. The forward algorithm first searches a small outlier-free subset and then iteratively refines the subset by adding one sample at a time. This is in contrast to the backward algorithms, which first deal with the entire data points and then delete bad samples. Fleishman et al. [35] showed that some outliers with large error may fail on fit based on the backward algorithms, whereas the forward algorithm gives satisfactory results. For our purpose, the initial subset is computed using the LMS algorithm using a small *k *value, where *k *is typically close to *p *for a model with *p *parameters (specially *p *= 3 in the 3D case) [35,39]. During the forward search, a number of parameters can be monitored to detect the influential points. Atkinson et al. [39] suggested several statistics, including the residual-plot, Cook's distance and others, to be monitored. For their purposes, these are plotted on a graph and inspected visually. In [35], Fleishman et al. suggested to monitor the maximal residual *r*_max_. The above monitoring techniques are essentially to determine the termination conditions for the forward search iteration. In our technique, we also monitor the maximal residual similar to Fleishman et al.'s strategy [35].

### The LMS fit algorithm

Using the forward search technique for solving Eq. (3), we present a new LMS fit algorithm for structural superposition of two point sets {**x**_*i*_} and {**y**_*i*_} with *N *points each in order to compute the centers and the rotation matrix **U**.

1. Compute the small outlier-free subset *Q*_x _⊆ {**x**_*i*_} and *Q*_y _⊆ {**y**_*i*_} using the LMS algorithm, which is described as *random sampling *above.

2. The centers and rotation matrix **U **are computed for *Q*_x _and *Q*_y _using the RMSD fit.

3. One pair of points with the minimal residual *r*_min _in the remaining point pairs are added into *Q*_x _and *Q*_y_, respectively.

4. Repeat steps 2 and 3 until *r*_min _is larger than a predefined threshold *r*_max _and the iteration number *iter *is larger than the minimal iteration number MIN_ITERS. Finally, identify points in *Q*_x _and *Q*_y _as the rigid core regions and points in ({**x**_*i*_} - *Q*_x_) and ({**y**_*i*_} - *Q*_y_) as outliers or flexible regions.

Implementation details of the LMS fit are described in **Appendix**.

#### Initial robust estimator

In the first step of the forward search algorithm, the initial subset is computed using the LMS algorithm with a small *k *value (we typically choose *k *= 3). If the atom number *N *of protein is small, the choice of the initial subset can be performed by exhaustive enumeration of all ($\left(\begin{array}{c} N\\ k \end{array}\right)$); otherwise, LMS uses the random sampling algorithm that requires a large iteration number *T *to achieve a high probability of finding a good estimator. The LMS algorithm, as a statistical method, assumes that the samples (points) are independent. If *g *is the probability of selecting a single good sample at random from two original point sets {**x**_*i*_} and {**y**_*i*_}, then the probability *P *of successfully finding *k *good samples after *T *iterations can be computed by *P *= 1 - (1 - *g*^*k*^)^*T *^[35]. In our implementation, we use *T *= 500 for the small proteins (e.g. *N *< 900) and *T *= 1000 for the large proteins (e.g. *N *≥ 900) in order to obtain both small errors and little computation time.

#### Termination conditions

In the fourth step, there are two termination conditions (i.e. *r*_min _> *r*_max _and *iter *> MIN_ITERS). *r*_max _is the threshold of maximal residual. The threshold *r*_max _controls the fitted subsets. Smaller values of *r*_max _does not use all of the available atom pairs to fit, while a larger value for *r*_max _requires more iterations and the algorithm becomes sensitive to outliers. If *r*_max _is too large such that the final subset is equal to the input point set, i.e. no outlier detected, the LMS fit is same to the RMSD fit algorithm. In some sense, the RMSD fit is only one special case of our algorithm. In our experiments, the errors would have to be on the order of Angstroms. We have found that *r*_max _in the range of 1Å to 4Å is able to highlight the similarity of the rigid core regions.

In addition, another termination condition, in which the iteration number *iter *should be larger than the minimal iteration number MIN_ITERS, is also very important. MIN_ITERS is usually chosen as a predefined integer to ensure that the number of atoms on core regions is more than 50% of atoms (generally [*N*/2.0] ≤ MIN_ITERS ≤ *N*). This constraint condition satisfies the LMS assumption in which the core regions contain at least 50% points of the entire point set. We typically choose MIN_ITERS as the half of the number of atoms, i.e. MIN_ITERS ⇐ [*N*/2.0].

#### A new similarity measurement

A standard RMSD fit minimizes the sum of residuals for entire atom pairs, whereas the LMS fit minimizes the median of residuals for entire atom pairs. When finishing the LMS fit using the forward search technique, we can obtain two similarity measurements. One is the median distance *D*_*median *_by computing Eq. (2) for two entire point sets. Another is the RMSD distance *D*_*rmsd *_by computing the the square root of Eq. (1) for the final subset *Q*_x _and *Q*_y_. Being different with *D*_*median*_and *D*_*rmsd *_defined by absolute distances, we present a new similarity measurement:

(4) $$\text{Core}\%=\frac{N_{\text{core}}}{N},$$

where *N*_core _is the number of atoms of the core region, and *N *is the number of entire atoms of protein. The value of Core% denotes the proportion of the core region (i.e. the final subset) that belongs to the entire point set. It is more intuitive to measure the similarity between two conformations than the absolute distances *D*_*median *_and *D*_*rmsd*_. The maximum value of Core% occurs when *N*_core _is equal to *N *(i.e. the distances between all atom pairs are less than *r*_max_). The lower the similarity, the smaller the value of Core%. The value of Core% can be directly used for the similarity score between two protein structures.

We will investigate the effect of Core% with respect to *r*_max _in the next section.

## Authors' contributions

YL generated the original idea, executed the research, and wrote the manuscript. YF participated in the research. KR supervised the project and edited the paper. All authors read and approved the final manuscript.

## Appendix: LMS implementation details

The outline of an algorithm for the LMS fit, called **LMSfit**, is given in Algorithm 1. The algorithm takes as input two point sets **X **and **Y **with *N *points each in order to compute the centers **c**_x _and **c**_y _and the rotation matrix **U**. This is achieved through an iterative procedure with the aid of two variables *Q*_x _and *Q*_y _which are the working subset of superposition between **X **and **Y**, respectively. Initially, *Q*_x _and *Q*_y _are computed using the LMS algorithm through selecting *k *point pairs at random with *T *iterations, as illustrated in Algorithm 2.

Algorithm 2 is passed into three point sets (**X **and **Y**) in order to produce *k *point pairs as the initial subset for the forward search (typically *k *= 3). First, a loop with *T *iterations begins. At each iteration, two subsets (*S*_x _and *S*_y_) with *k *points each are selected at random, and then two centers of the two subsets and a rotation matrix are computed using the standard RMSD fit. Next, residuals of all point pairs in the remaining subsets are calculated as the distance between each rotation point and the corresponding target point. Finally, the median *r*_median _of residuals of the remaining subset is obtained. The subsets (*S*_x _and *S*_y_) with the minimal median *r*_median _are returned as the final initial subsets (*Q*_x _and *Q*_y_), respectively. During the iterative procedure in Algorithm 1, the cardinality of *Q*_x _and *Q*_y _is gradually increased by adding one pair of points (x* and y*) with the minimal residual every time. In this way, one is able to increase *Q*_x _and *Q*_y _regarded as the core region in the forward search. If the residuals of the remaining point pairs are more than a threshold *r*_max_, the procedure is terminated. Finally, the final subset *Q*_x _and *Q*_y _are regarded as the core regions and the points in *Q*_x _and *Q*_y _are used to compute the final centers **c**_x _and **c**_y _and the rotation matrix **U**, and the remaining points are identified as outliers or flexible regions.

**Algorithm 1**: **LMSfit**(**X**, **Y**, **c**_x_, **c**_y_, **U**)

Input: **X**: the first point set with *N *points

**Y**: the second point set with *N *points

Output:

**c**_x _and **c**_y_: the final centers of **X **and **Y**

**U**: the rotation matrix

Local variables:

*k*: the number of random samples

*Q*_x _and *Q*_y_: the subsets of **X **and **Y**

*R*_x _and *R*_y_: the sets of the remaining points, i.e. *R*_x _⇐ **X **- *Q*_x _and *R*_x _**Y **- *Q*_y_

${\tilde{c}}_{\text{x}}$ and ${\tilde{c}}_{\text{y}}$: the temporary centers

$\tilde{U}$: the temporary rotation matrix

*r*_min_: the minimal residual

*r*_max_: the maximal residual

begin

1: *Q*_x _⇐ ∅; *Q*_y _⇐ ∅;

2: **LMS**(**X**, **Y**, *k*, *Q*_x_, *Q*_y_);

3: *I *⇐ 0;

4: *R*_x _⇐ **X **- *Q*_x_; *R*_y _⇐ **Y **- *Q*_y_;

5: MIN_ITERS ⇐ [*N*/2.0];

6: **while **(|*R*_x_| > 0) **do**

7:    Compute two centers ${\tilde{c}}_{\text{x}}$ and ${\tilde{c}}_{\text{y}}$ for *Q*_x _and *Q*_y_;

8:    Translate **X **and **Y **to ${\tilde{c}}_{\text{x}}$ and ${\tilde{c}}_{\text{y}}$, and compute the rotation matrix $\tilde{U}$ for two translated point sets using the standard RMSD fit algorithm;

9:    /* Compute **r **as residuals of all pairs of points between *R*_x _and *R*_y _*/

10:    **for **(*i *= 0; *i *< |*R*_x_|; *i *+ +) **do**

11:       **x**_*i *_⇐ *R*_x_(*i*) and **y**_*i *_⇐ *R*_y_(*i*);

12:       **r**(*i*) ⇐ ||$\tilde{U}$(**x**_*i *_- ${\tilde{c}}_{\text{x}}$) - (**y**_*i *_- ${\tilde{c}}_{\text{y}}$)||;

13:    **end for**

14:    Get the pair of points x* and y* with the minimal residual *r*_min _for **r**;

15:    **if **(*r*_min _> *r*_max _and *I *> MIN_ITERS) **then**

16:       **return**

17:    **end if**

18:    /* Update the subsets and the remaining point sets */

19:    *Q*_x _⇐ *Q*_x _+ x* and *Q*_y _⇐ *Q*_y _+ y*;

20:    *R*_x _⇐ *R*_x _- x* and *R*_y _⇐ *R*_y _- y*;

21:    /* Update the centers and rotation matrix */

22:    **c**_x _⇐ ${\tilde{c}}_{\text{x}}$, **c**_y _⇐ ${\tilde{c}}_{\text{y}}$, and **U **⇐ $\tilde{U}$;

23:    *I *+ +;

24:    **end while**

end

**Algorithm 2**: **LMS**(**X**, **Y**, *k*, *Q*_x_, *Q*_y_)

Input:

**X**: the first point set with *N *points

**Y**: the second point set with *N *points

*k*: the number of random samples

Output:

*Q*_x _and *Q*_y_: the initial subsets from **X **and **Y**

Local variables:

*T*: the number of iterations

*S*_x _and *S*_y_: the subsets selected randomly

*R*_x _and *R*_y_: the set of the remaining points, i.e. *R*_x _⇐ **X **- *S*_x _and *R*_y _⇐ **Y **- *S*_y_

**r**: the vector of redsiduals

**c**_x _and **c**_y_: the centers of the subsets *S*_x _and *S*_y_

**U**: the rotation matrix

*r*_median_: the median of redsiduals

*r*_min_: the minimal redsidual

begin

1: *r*_min _⇐ ∞;

2: **for **(*j *= 0; *j *<*T*; *j *+ +) **do**

3:    Select randomly *k *pairs of points: *S*_x _and *S*_y_, with the same order from **X **and **Y**, respectively;

4:    Compute two centers **c**_x _and **c**_y _for *S*_x _and *S*_y_;

5:    Translate *S*_x _and *S*_y _to **c**_x _and **c**_y_, and then compute the rotation matrix **U **for two translated subsets using the RMSD algorithm;

6:    Compute the sets of the remaining points as: *R*_x _⇐ **X **- *S*_x _and *R*_y _⇐ **Y **- *S*_y_;

7:    /* Compute **r **as residuals of all pairs of points between *R*_x _and *R*_y _*/

8:    **for **(*i *= 0; *i *< |*R*_x_|; *i *+ +) **do**

9:       **x**_*i *_⇐ *R*_x_(*i*) and **y**_*i *_⇐ *R*_y_(*i*);

10:       **r**(*i*) ⇐ ||**U**(**x**_*i *_- **c**_x_) - (**y**_*i *_- **c**_y_)||;

11:    **end for**

12:    Compute the median *r*_median _by sorting **r**;

13:    **if **(*r*_median _<*r*_min_) **then**

14:       *r*_min _⇐ *r*_median_;

15:       *Q*_x _⇐ *S*_x _and *Q*_y _⇐ *S*_y_;

16:    **end if**

17: **end for**

end
